# Human Breast Milk Promotes the Immunomodulatory Function of Probiotic *Lactobacillus reuteri* DSM 17938 in the Neonatal Rat Intestine

**DOI:** 10.35248/2329-8901.19.7.210

**Published:** 2019-07-26

**Authors:** Thomas K Hoang, Jasmin Freeborn, Ting Wang, Tu Mai, Baokun He, Sinyoung Park, Dat Q Tran, Stefan Roos, J Marc Rhoads, Yuying Liu

**Affiliations:** 1 Department of Pediatrics, The University of Texas Health Science Center at Houston McGovern Medical School, Houston, Texas 77030, United States; 2 Department of Molecular Sciences, Uppsala BioCenter, Swedish University of Agricultural Sciences, Uppsala, Sweden

**Keywords:** Probiotic, *Lactobacillus reuteri*, Neonatal rats, T cell, Dendritic cell, Formula, Human breast milk, Intestine

## Abstract

**Background and objective:**

Breast milk has many growth-promoting and immune-active components, including transforming growth factor-β, lactoferrin, lysozyme, immunoglobulin A, and prebiotics such as the human milk oligosaccharides. Treatment with *Lactobacillus reuteri* DSM 17938 (LR), a probiotic with immunomodulatory functions, significantly increases regulatory T cells (Tregs) in the intestinal mucosa of newborn suckling rats. In humans, treatment with LR of infants with colic reduces crying optimally if the infants are breast-fed. Therefore, we examined the effects of human breast milk (HBM) on LR-associated immune modulation.

**Methods:**

Newborn rats were divided into 8 feeding groups, including dam-fed ± LR (10^6^ CFU/kg bw/day, daily), formula-fed ± LR, formula with 20% (v/v) HBM-fed ± LR, and HBM-fed ± LR. Pups were fed by gavage from d1 to d3 of age. Subsequently, we measured intestinal immune cell profiles, including Tregs and tolerogenic dendritic cells (tDCs) by flow cytometry. We also measured inflammatory cytokine and chemokine levels of interleukin (IL)-1β and cytokine-induced neutrophil chemoattratant (CINC)-1 in intestinal tissue lysates by ELISA.

**Results and Conclusion:**

(1) Formula feeding increased intestinal CD3+ T cells, CD4+ helper T (TH) cells and CD11c+ DCs, pro-inflammatory effects which were reversed by HBM. (2) When comparing HBM-fed with formula-fed newborns, HBM supplementation produced a lower percentage of CD4+ TH cells and a higher percentage of CD8+ (cytotoxic) T cells, while reducing protein levels of IL-1β and CINC-1 in the intestine. (3) Probiotic LR feeding maximally stimulated the percentage of intestinal Tregs and tDCs when the pups were fed HBM. In conclusion, HBM reduced formula-induced intestinal gut immune activation, and the addition of LR further promoted immune tolerance.

## INTRODUCTION

*Lactobacillus reuteri* DSM 17938 (LR) is a probiotic bacteria originally isolated from a Peruvian mother’s breast milk [1,2]. LR inhibits pathogen growth and modulates the immune system. In humans, LR is under investigation among others for preventing necrotizing enterocolitis (NEC) in premature infants [3,4], for reducing the severity of acute infant diarrhea [5,6] and for decreasing crying time in infants with colic [7].

Immunomodulatory activities of LR paradoxically include a mild pro-inflammatory effect in monocytoid [8] and intestinal epithelial [9] cells *in vitro,* counterbalanced by strong overall anti-inflammatory effect *in vivo* animal models of NEC [10–12]. We have found that feeding LR significantly reduced the incidence and severity of NEC *via* a modulation of pro-inflammatory signaling *via* Toll-like receptor 4 (TLR4) and nuclear factor-B (NF-B), resulting in decreased mucosal pro-inflammatory cytokines such as tumor necrosis factor (TNF)-α and interleukin (IL)-1β [11].

LR modulates the composition of intestinal immune cells in neonatal gut, including dendritic cells (DCs), effector memory T (Tem) cells (eg. TH1/TH2/TH17 cells), and regulatory T (Treg) cells. Tregs are a distinct T cell subpopulation that is engaged in sustaining immunological self-tolerance and homeostasis. The transcription factor FoxP3 plays a key role in Treg development and function [13]. Tregs regulate and attenuate other TH subsets to control inflammation and allergic responses. In the absence of Tregs, Tem cells lose control and migrate to peripheral sites, where they produce inflammation. For example, the scurfy mouse is a model of a multi-organ human disease caused by Treg-deficiency, called IPEX syndrome (immunodysregulation, polyendocrinopathy enteropathy, with X-linked inheritance) syndrome [14,15]. LR prolongs survival and reduces disease severity of scurfy mice by modulating gut microbiota, profoundly altering a number of key metabolites, and inhibiting TH1/TH2 development *via* the adenosine receptor 2A [16,17].

In neonatal NEC models, we found that LR facilitates the induction of immune-modulating Tregs while lowering pro-inflammatory Terns in the intestinal mucosa [11,12]. We recently reported that the protective effect of LR against NEC (involving DCs and T cells) is mediated by TLR2 signaling [18]. DCs are central to this process. Tolerogenic DCs (tDCs) facilitate the generation of tolerogenic T cells (such as Tregs) recognizing food antigens and the commensal microbiota and preventing unnecessary hypersensitivity reactions [19].

During our NEC studies, we observed that LR combined with suckling (breast milk) in newborn rat pups significantly increased the proportion of Tregs in the intestine; and a change in Tregs was observed within 24 hours. This phenomenon was much slower in the formula-fed animals that were separated from their mothers immediately after birth [10]. These results imply that breast milk components may enhance immune modulation in response to LR. Breast milk contains lactose, whey, casein, and a variety of bioactive components such as IgA, lactoferrin, β -lactoglobulin and α-lactobumin that directly or indirectly modulate the immune system [20]. Maternal milk is also the major source of epidermal growth factor (EGF) for neonates, which plays an important role in preventing NEC [21]. Human milk oligosaccharides, the major carbohydrate in breast milk, powerfully facilitate the colonization by beneficial Bifidobacteria in the newborn [22]. However, it is unknown whether human breast milk (HBM) promotes the immunomodulatory activity of LR in the neonatal gut.

In this study, we examined the effects of LR under different feeding conditions in the newborn rat, including dam-feeding, cow-milk-based formula feeding, feeding cow-milk formula supplemented with 20% (vol/vol) of HBM (20% HBM), and finally HBM (100% HBM) feeding. We measured the impact of milk source on T cells (total T cell number, TH cells, cytotoxic T cells, and Tregs), tDCs and quantified changes in pro-inflammatory signaling proteins including cytokine interleukin (IL)-1β and chemokine cytokine-induced neutrophil chemoattractant (CINC)-l) in the intestines of newborn rats. Our observations provide evidence that human breast milk modulates the immunological effects of probiotics in the neonatal gut.

## MATERIALS AND METHODS

### Rats and experimental design

All *in vivo* experiments were performed on newborn Sprague-Dawley rat pups (Harlan Laboratories, Indianapolis, IN) weighing 5-6 g. Newborn rat pups were either allowed to stay with their dams (dam-fed group) or were separated from their dams on day 1 of age (d1), housed in an incubator, and fed by gavage with either formula, formula+20% (v/v) HBM or 100% HBM 5 times daily (7 am-11 am-3 pm-7 pm-11 pm) for 3 days.

The formula used was cow milk based mixture of Abbott Similac PM60/40 low-iron infant formula (15 g) in 75 mL of Pet-Ag Esbilac puppy milk replacer. We fed by gavage 10^6^ colony-forming unit (CFU)/g bw/daily to designated groups of rats. HBM was collected by breast pump and mixed, fresh-frozen at −20°C for less than 1 month before being fed to rats. On d4 of age, intestinal tissues were collected; immune cells were isolated for cell analysis from parts of fresh tissues. Fresh frozen tissues were stored at −80°C for further tissue lysates used for measuring IL-1β and CINC-1 levels by ELISA.

All rats were housed in the specific pathogen free animal facility at the University of Texas Health Science Center at Houston. This study was carried out in accordance with the recommendations of the Guide for the Care and Use of Laboratory Animals (NIH), the Institutional Animal Care and Use Committee (IACUC). The protocol was approved by the IACUC (protocol numbers: AWC-14-064).

### Probiotic LR preparation

LR obtained from BioGaia AB (Stockholm, Sweden) [2] was anaerobically cultured in deMan-Rogosa-Sharpe (MRS; Difco, Detroit, MI) medium at 37°C for overnight. To generate a standard curve for bacterial growth for quantitative analysis of bacteria in culture media, we plated evenly with 100 μL of bacterial culture in MRS agar plates at specific serial dilutions, followed by anaerobically culturing at 37°C for 48-72 h to count bacterial colonies. Finally, the bacterial CFU/mL in MRS medium were calculated. A standard curve was generated by comparing absorbance at 600 nm of cultures to the known CFU/mL under serial dilutions. Quantitative analysis of bacteria in culture media was calculated based on the standard curve by measuring the absorbance of 600 nm (Photometer, Eppendorf, Hamburg, Germany). Bacteria in the culture media were harvested by centrifugation at 1500 × g for 15 min and resuspended in certain volumes of formula before feeding.

### Tissue preparation for flow cytometry

Collected intestinal tissue (ileum) was incubated for 30 minutes at 37°C in PRMI-1640 (Sigma) complete medium containing collagenase V from Clostridium histolyticum (Sigma, St. Louis, MO) at the concentration of 0.1 mg/mL followed by vigorously vortexing for 1 min, single-cell suspensions were obtained by filtration through 40-μm cell strainers (BD Biosciences, San Jose, CA). Cells were washed with MACS buffer consisting of phosphate-buffered saline, 0.5% bovine serum albumin (Hyclone Laboratories), and 2 mM EDTA (Lonza), and finally re-suspended in MACS buffer performing surface and intracellular staining using specific antibodies for further flow cytometric analysis.

### Flow cytometric analysis

Cells were detected by immunofluorescent staining with flow cytometric analysis. Cell surfaces were stained by fluorochrome-conjugated anti-rat antibodies, T cell panels included CD3 (G4.18), CD4 (OX-35), CD8a (OX-8) from BD Pharmingen (San Diego, CA). Intracellular staining for Foxp3 was performed with fixation/permeabilization kit according to the manufacturer’s protocol (eBioscience, San Diego, CA) and detected with anti-Foxp3 (150D, BioLegend, San Diego, CA). DC panels included CD11b/c (OX-42), RT1D (MHC II, OX-17), CD103 (OX-62) from BioLegend. All samples were analyzed with BD FACSCalibur. Further, data were analyzed by using Flowjo software.

## Cytokine IL-1 β and chemokine CINC-1 assays

Ileal tissues were homogenized with 0.4 mL of RIPA lysis buffer (Cell Signaling Technology) mixed with a protease inhibitor cocktail (Sigma). After homogenizing, tissues were centrifuged at 13,200 g for 10 min at 4°C and the supernatants were collected. A DC protein assay (Bio-Rad) was performed to measure total protein in the tissue lysates by mg/mL using bovine serum albumin (BSA) as a standard. Rat ELISA cytokine kits (R&D Systems) were used according to manufacturer’s protocol to measure IL-1 β and CINC-1 levels in the collected samples. Cytokine levels measured were normalized by the concentrations of total protein measured in homogenized supernatants of ileal tissues and were reported as pg of IL-1 β or CINC-1 per mg total protein.

## Statistics

Experimental results were expressed as means ± SD. Statistical analysis was performed using one-way ANOVA (Graph Pad Prism 4.0; GraphPad Software, San Diego, CA). Dunnett’s and Tukey’s multiple-comparison tests were used for comparison of multiple groups with a control group or multiple groups. p value of <0.05 was considered statistically significant.

## RESULTS AND DISCUSSION

### Different feedings shaped intestinal T cell populations in neonatal rats

Following birth, environmental factors, nutritional constituents, and the rapidly emerging microbiota facilitate the maturation of the mucosal immune system. T lymphocytes generated in the thymus populate secondary lymphoid organs and generate a broad spectrum of adaptive T-cell immunity that ultimately plays a critical role in the establishment of life-long host-microbial homeostasis and antimicrobial host defense [23]. We measured the proportions of T cells including CD4+ TH and CD8+ cytotoxic T cells in the intestine and mesenteric lymph nodes of 4 days of age (d4) rats after receiving 3-days of different diets, starting on d1. We stained cells by antibodies recognizing T cell CD markers and analyzed the lymphocyte populations by using flow cytometry. The gating strategy to identify specific T cell populations including CD3+ T, CD4+ TH, CD8+ cytotoxic T and Foxp3+Treg cells is indicated in Supplemental Figure 1.

(1) Feeding source differentially regulated intestinal CD3+ T cell, CD4+ TH, and CD8+ cytotoxic T cells in neonatal rats.

CD3 is a T cell marker required for activation. We found that the percentage (mean ± SD) of intestinal CD3+ T cells among total lymphocytes was 7.6 ± 4.7% in dam-fed newborn rats, while it was significantly increased in neonatal rats fed with formula (23.9 ± 6.3%) (p<0.001).

**Figure 1:**
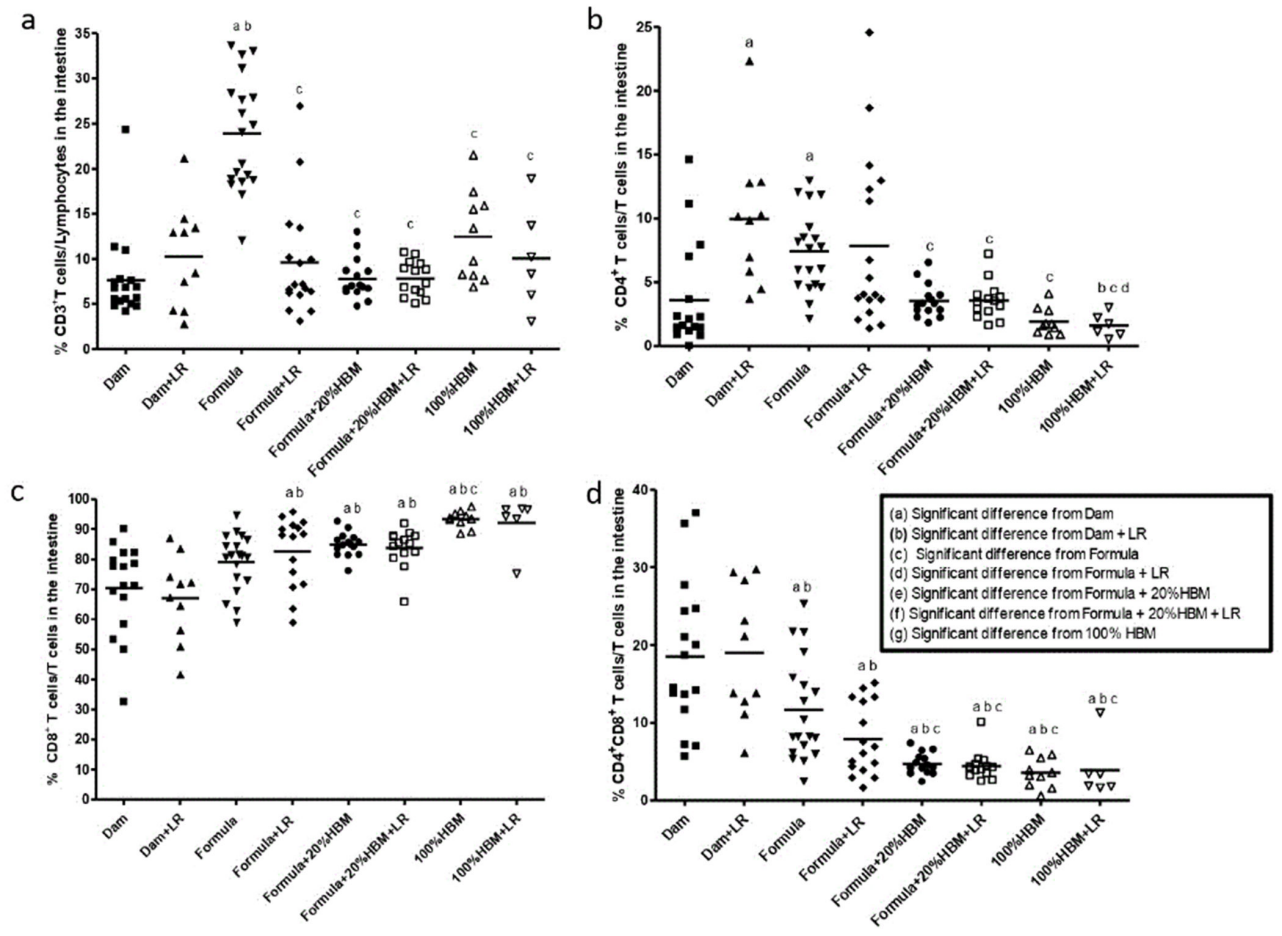
Frequencies of T cell subsets: (a) CD3+, (b) CD4+, (c) CD8+, and (d) CD4+CD8+ DP amongst cells in the ileum of newborn rats exposed to different feeding regimens. In each feeding group, a dot represents one rat. The line in each group represents the mean value. Animal numbers in groups were n=6-19. Multiple group comparisons were performed using one way ANOVA with post hoc Tukey’s test. The multiple group comparisons with significant p values (p<0.05,<0.01, or<0.001) are indicated in the Figures.

The increase could be reduced by supplementation with either (a) probiotic LR, adding 20% of HBM to the formula, or (b) feeding HBM (100% HBM) (all p<0.001) (Figure 1a). Further analyzing CD4+ TH cells among these T cell population, we observed that feeding dam-fed newborn rats with LR increased TH cells in the rat intestine (p<0.01). Compared to dam-fed pups, formula also increased the proportion of TH cells (p<0.05), an effect which could be decreased by adding 20% HBM to the formula or feeding by gavage 100% HBM, either in the presence or absence of LR (all p<0.05) (Figure 1b). In addition, LR in HBM significantly reduced TH cells in the intestine compared to feed LR in dam-fed rats (p<0.01) or compared to feed LR in formula to rats (p<0.05). This finding indicates that HBM is different from rat breast milk or formula in allowing LR to modulate the immune system (Figure 1b). Analysis of CD8+ cytotoxic T cells revealed that the percentage (mean ± SD) of intestinal CD8+ T cells among the T lymphocyte population was 70.4 ± 15.1% in dam-fed rats, which was significantly increased in rats receiving either formula containing 20% HBM (84.8 ± 3.9%) or 100% HBM (93.3 ± 2.8%) (p<0.01), regardless of the presence of LR (all p<0.001). CD8+ T cells were also increased in response to HBM feeding compared to formula feeding (p<0.05). Compared to feeding LR to dam-fed rats, feeding LR to formula, or to formula plus 20% HBM or to HBM increased the% of CD8+ T cells in the intestine of rats (Figure 1c). We observed high proportions of CD8+ T cells and CD4+CD8+ doubly positive (DP) T cell populations in the intestine of dam-fed rats. This finding was specific for rats but was not previously found in mice [10,12]. Finally, we found that formula and HBM (both 20% and 100%) without influenced by LR reduced the proportion of intestinal CD4+CD8+ DP T cells in neonatal rats (Figure 1d). These findings in our studies show that human breast milk can reduce and modulate formula-induced intestinal gut immune activation in the early days of the newborn rat’s life.

(2) Human breast milk promoted the modulation of LR on Tregs in the intestine of neonatal rats.

**Figure 2:**
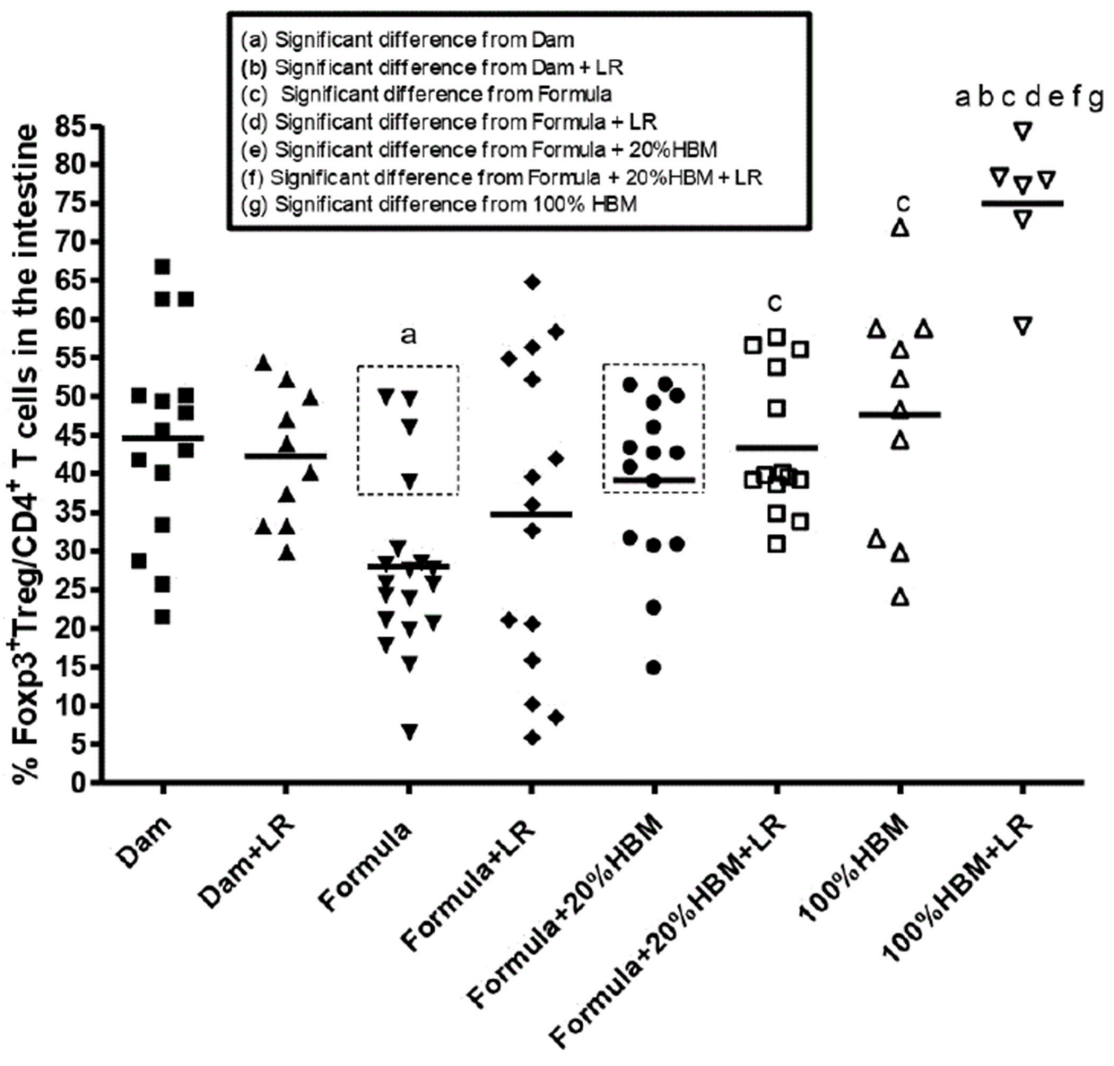
Percentages of Foxp3+ LReg cells among CD4+ T cells in the ileum of newborn rats exposed to different feeding regimens. In each feeding group, a dot represents one rat. The line in each group represents the mean value. Animal numbers in groups were n=6-19. Multiple group comparisons were performed using one way ANOVA with post hoc Tukey’s test. The multiple group comparisons with significant p values (p<0.05,<0.01, or<0.001) are indicated in the Figures. Dotted rectangles indicate the numbers of rats with % of LRegs greater than or equal to the mean % of LRegs in Formula+20% HBM-fed rat pups.

Amongst CD4+ TH cells, Foxp3+ Treg cells are the master regulators, which attenuate other TH subsets to induce self-tolerance and control inflammation [24]. We focused on intestinal Foxp3+ Tregs (Figure 2). We found that formula-feeding significantly reduced the % of Tregs compared to dam-feeding (p<0.01). Feeding rats with HBM increased the percentage of Tregs by about 8% in formula-fed subjects (p<0.01). Comparing the groups of formula-fed mice with formula+20% HBM mice, 10 out of 15 mice in the formula +20% HBM group had an increase the mean value of Tregs; however, only 4 out of 19 mice that were exclusively formula-fed had an increase in mean value of % Tregs (dotted rectangles in Figure 2). These results indicate that human breast milk (whether amounting to 20% or 100% of the milk supplied) facilitates the generation of intestinal Tregs.

Previous studies indicated that when LR was fed to newborn dam-fed rats, it increased the % of intestinal Tregs almost immediately at d1 and d2 of age. Subsequently, the % of gut Tregs rose to similar level between two groups at d3 and d4 of age (reaching a level of ~40% of total TH cells). This phenomenon was observed in dam-fed rats but not in formula-fed pups [10], indicating that there may be a synergistic effect between breast milk and LR. In the present study, results indicated that LR fed dam-fed rats did not change the % Tregs at d4 of age of rats compared to dam-fed rats without LR feeding (44.5 ± 13.5% *vs.* 42.2 ± 8.6%). When all subjects were analyzed collectively, LR-fed formula-fed rats also did not result in an increase in the % of Tregs compared to formula-fed only rats (34.7 ± 19.9% vs. 27.9 ± 11.4%). However, addition of 20% HBM to formula while feeding LR increased the % of Tregs in the intestine of rats compared to formula alone (43.4 ± 9.1% *vs.* 27.9 ± 11.4%, p<0.05). The most pronounced effect was seen when comparing rats fed with 100% HBM; in this group, feeding LR powerfully increased % of Tregs to 75.0 ± 8.6% in the intestine. Compared to formula+LR,; to formula+20% HBM+LR, or to 100% HBM, this result was quite significant (all p<0.01) (Figure 2). These results indicated that human breast milk strongly promotes the effect of LR on intestinal Tregs.

Recent studies have demonstrated that milk could stabilize Foxp3 expression and Treg differentiation [25]. This process is mediated by amino acids in the milk, such as essential branched-chain amino acids (promoting the secretion of insulin) and tryptophan (increasing the secretion of hepatic insulin-like growth factor-1 (IGF-1)); by milk-enriched mRNAs and microRNAs; and by exosomal transforming growth factor-β (TGF-β ). Amino acids and growth hormone were found to synergistically activate the phosphoinosite-3 kinase (PI3K)-Akt and the nutrient-sensitive kinase mechanistic target of rapamycin complex 1 (mTORC1). The Akt-mTORC1 axis controls Foxp3 expression; thus, a well-balanced transfer of critical amino acids *via* breastfeeding appears to control Akt-mTORC1-mediated Treg differentiation. Formula, conversely, contains proteins, which may disturb the Akt pathway activation [25]. Human breast milk is also enriched in microRNAs, which promote DNA demethylation at the Treg-specific demethylated region (TSDR) to stabilize Foxp3 expression. Finally, milk-derived exosomal TGF-β activates the transcriptional factor SMAD3 to enhance Foxp3 expression *via* binding to the conserved non-coding sequence 1 (CNS1) of Foxp3 [26]. All of this evidence indicates that milk promotes the stabilization of Foxp3 and promotes long-lasting Treg differentiation, crucial in preventing atopic and autoimmune diseases postnatally.

### Different feedings affected intestinal dendritic cells (DCs) in neonatal rats

**Figure 3:**
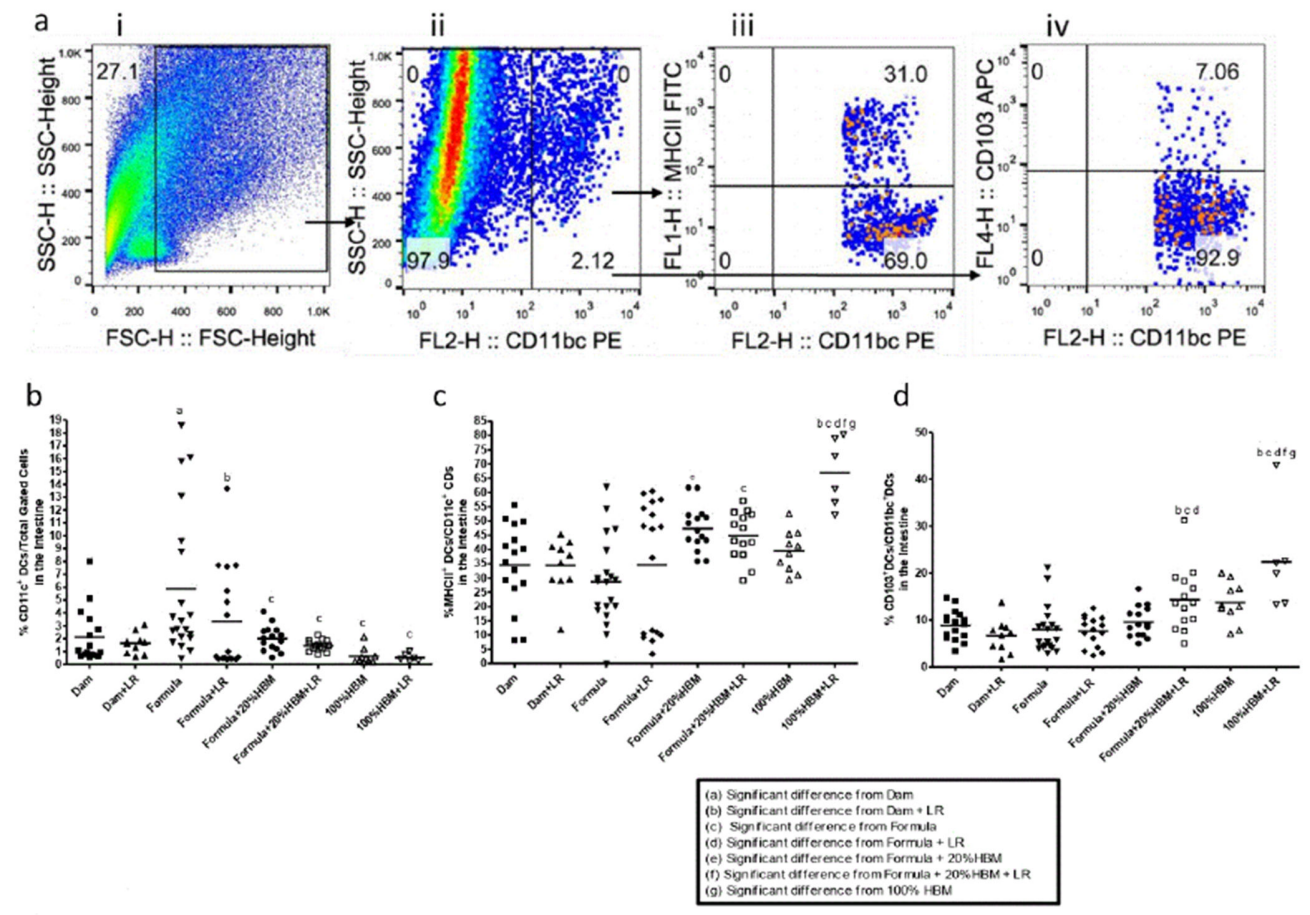
Frequency of different classes of DC’s, (a) Gating strategy to define cell populations. Representative flow cytometric plots from ileum samples of dam-fed were initially gated on the “ whole ” population (i) on a forward scatter (FSC)-side scatter (SSC) plot, followed by CD11b/c+ dendritic cells (DCs)(ii). Subsequently, MHCII +DCs were identified (iii), and differentiated CD 103+ DCs were quantified (iv). Frequency of (b) CD11b/c+ dendritic cells; (c) MHCII+ DCs, and (d) CD 103+ tolerogenic DCs in the ileum of newborn rats with different feeding regimens. In each feeding group, each dot represents a single rat pup. The horizontal line in each group represents the mean value. Animal numbers in groups, n=6-19 pups. Multiple group comparisons were performed using one way ANOVA and post hoc Tukey’s test. The multiple group comparisons with significant p values (p<0.05,<0.01, or<0.001) are indicated in the Figures.

Probiotics support mucosal immunity in ways that are similar to the commensal bacteria in the human gut. They interact with DCs to induce DC maturation, with the primed tolerogenic DCs being capable of driving the development of Treg cells [27]. We defined cell populations of intestinal DCs by flow analysis as follows: total dendritic cells (CD11c+), mature DC (MHC II+), and tolerogenic DCs (tDCs) (CD103+). Results are shown in Figure 3a.

Our studies showed that intestinal CD11c+ DCs increased in formula-fed newborn rats compared to dam-fed rats (p<0.05). Human breast milk (either 20% HBM added to formula (p<0.01) or 100% HBM (p<0.001)) reduced formula feeding-associated increased DCs in the intestine of rats with either in the presence of absence of LR (Figure 3b). Adding TR to the formula did not further reduce the% of DCs in the intestine (Figure 3b).

However, addition of 20% HBM to formula increased MHCII+ DCs in the intestine of rats compared to rats fed formula only (p<0.01). Interestingly, feeding LR in HBM significantly boosted MHCII+ DCs compared to rats fed with HBM alone (p<0.01), with the highest levels compared to all other groups. Apparently, LR in HBM significantly increased MHCII+ DCs compared to LR fed to dam-fed rats (p<0.001), to formula-fed rats (p<0.01), or to formula+20% HBM rats (p<0.05) (Figure 3c).

Besides priming T cell differentiation, mature DCs can limit effector T cell responses and promote immune tolerance [28]. The subset of DCs with steady-state tolerogenic properties is defined as the population of CD 103+ DCs present into the intestinal mucosa, DC’s that play a central role in enforcing tolerance to commensal bacteria and food antigens [29]. We examined CD 103+ DCs in the intestine of newborn rats. We observed that feeding LR to formula+20% HBM-or to HBM-fed rat pups significantly increased the percentage of intestinal CD103+ DCs. In addition, LR feeding to 100% HBM-fed pups enhanced the% of CD 103+ DCs compared to 100% HBM alone (Figure 3d).

Intestinal resident tDCs (CD11c+CD103+ DCs) play an important role in the process of Treg differentiation [30]. They are required for the induction of tolerogenic immune responses and contribute to the control of inflammatory responses and homeostasis in the intestinal mucosa by orchestrating the conversion of naive T cells into Foxp3+ Tregs [29]. Functionally specialized CD103+ DCs derived from the small intestinal lamina propria appear to be the only cells able to regulate T cell homing [31]. Whether intestinal DCs act to allow tolerance (as tDCs) or to enhance immunity (inflammatory DCs), gut microbiota are believed to have a key role-via direct action (microbe-DCs) and/or indirect interactions (microbe-epithelium-cytokine-DCs) [32]. We observed that both human breast milk and LR reduce the percentage of total DCs in the intestinal mucosa, while more importantly interacting with each other to increase tDCs in the intestine.

### Human breast milk and *Lactobacillus reuteri* modulate immune responses

Compared to formula feeding, breastfeeding has long been associated with lower infant morbidity and mortality, as well as with reduced incidence and severity of infections and immune-related diseases. Human breast milk beneficially influences neonatal gut microbiota acquisition, which represents a crucial stage in gut maturation, metabolic and immunologic programming [33]. In addition to the wide range of bioactive components, including digestive and antioxidative enzymes, growth factors, antimicrobial compounds, antibodies and cytokines, human breast milk is a source of viable commensal maternal bacteria capable of colonizing the neonatal gut. Both Bifidobacteria and Lactobacilli isolated from human breast milk have been studied and shown to facilitate Foxp3-LReg responses in the small intestine and to exert anti-inflammatory intestinal immune responses [33].

**Figure 4:**
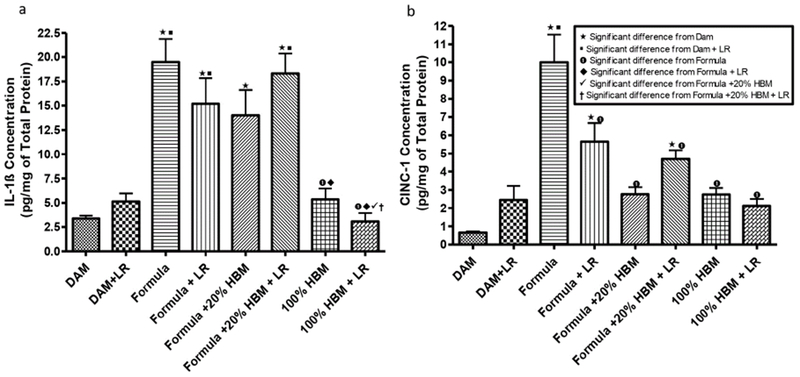
Levels of IL-1β and CINC-1 in intestinal tissue lysates of newborn rats exposed to different feeding regimens. IL-1 β and CING1 in the intestinal tissue lysates were detected by using ELISA. The concentrations of IL-1 β and CINC-1 were normalized by total protein levels in the intestinal tissue lysates, expressed as pg/mg total protein. Animal numbers in groupos were n=6-19. Multiple group comparisons were performed using one way ANOVA with post hoc Tukey’s test. The multiple group comparisons with significant p values (p<0.05,<0.01, or<0.001) are indicated in the Figures.

In our study, we quantified levels of IL-1 β , a pro-inflammatory lymphocyte-activating cytokine [34], and CINC-1, a leukocyte-aggregating chemokine which has shown to mediate inflammatory pathways by the release of amines [35]. We measured levels of these two cytokines in the intestines of newborn rats subjected to different sources of nutrition at birth. The data showed significance differences in between the groups that each have unique sources of milk or null, with or without LR (Figure 4). Levels of IL-1 β significantly increased in formula fed newborns when compared to dam-fed controls (Figure 4a) (p<0.001). Intervention with 100% HBM, with or without LR produced a significant reduction of IL-1 β compared to newborns solely formula-fed (P<0.001). In addition, we observed that LR in 100% HBM significantly reduced IL-1 β level compared to LR in 20% HBM. Formula feeding increased levels of CINC-1 in the intestine (Figure 4b) when compared to dam-fed controls, whereas HBM (20% HBM or 100% HBM) reduced the formula-increased levels of CINC-1 (p<0.001). In addition, LR, when was supplemented in formula, was able to decrease formula-induced CINC-1 levels (p<0.01). The results indicate that HBM and LR supplementation have the capacity to modulate the cytokine responses that occur with formula feeding.

The mechanisms by which human breast milk accentuates immune modulation in response to LR are still undefined and may be multifactorial. Human breast milk harbors structurally diverse, non-digestible oligosaccharides (human milk oligosaccharides, HMOs) that can enhance the growth of beneficial bacteria, such as Bifidobacteria [36]. We have found that LR when cultured grows better in human breast milk than in cow-milk based formula [37]. Animal milk (cow milk or goat milk) contains much less oligosaccharides than human milk, even though goat milk contains five to eight times higher levels of oligosaccharides (3-sialyllactose, 6-sialyllactose, disialyllactose, N-glycolylneuraminyllactose, 3-galactosyllactose, N-acetylglucosaminyllactose, and lacto-N-hexose) than cow milk [38]. For rat milk, a study showed that it contains 10-times higher protein levels and greater amounts of glycerophosphocholine and phosphocholine than human milk [39]. However, no data currently are available for oligosaccharides in rat milk. How HMOs and/or other factors in human breast milk facilitate LR growth requires further investigation. Human breast milk may also promote LR ‘ s interaction with other gut bacteria, thereby indirectly, modulating immune homeostasis in the intestine.

## CONCLUSION

Human breast milk reduces the inflammatory milieu (immune cells and cytokines/chemokines) in the intestine of newborn rat pups caused by consumption of cow milk-based formula. It makes biological sense that a probiotic such as LR would adapt best when given with the human milk that was its original source. However, it is even more interesting that its biological function (immune modulation) is enhanced when administered with human milk. These findings have led us to begin investigating the metabolic products of LR grown in different human milk samples [40]. Some of these products may have hitherto unknown properties on the innate intestinal immune system.

## Supplementary Material

1
