# Supplementary material for: Human Breast Milk Promotes the Immunomodulatory Function of Probiotic Lactobacillus reuteri DSM 17938 in the Neonatal Rat Intestine
Source: J Probiotics Health. Author manuscript; Available in PMC 2019 Sep 27. (PMC6764460; doi:10.35248/2329-8901.19.7.210)
Supplement: 1 [file NIHMS1044803-supplement-1.pdf]

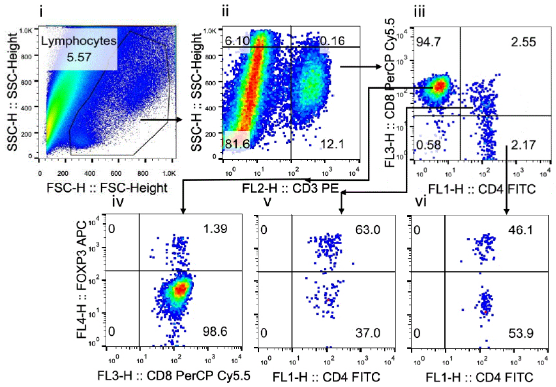

Supplementary Figure 1: Gating strategy to define cell population. Representative flow cytometric plots from ileum of dam-fed were initially gated as "lymphocyte" populations (i) on forward scatter (FSC)-side scatter (SSC) plot, followed by CD3+ T cells (ii), subsequently they were gated as CD4+ T (T helper, TH) cells (x-axis), CD8+ T (cytotoxic T) cells (y-axis), and CD4+CD8+ double positive (DP) T cells (iii). Finally, Foxp3+ Treg cells within the group of CD4-CD8+ (iv), or of CD4+CD8- (v), or of CD4+CD8+DP (vi) were identified and quantified.
